# 1339. Effects of Tixagevimab-Cilgavimab in Clinical Practice is Consistent with Preliminary Neutralization Studies

**DOI:** 10.1093/ofid/ofad500.1176

**Published:** 2023-11-27

**Authors:** Daniela Dluzynski, Paddy Ssentongo, Shareef Shaheen, Natella Maglakelidze, Cory Hale, Maria Paula Henao, Vernon M Chinchilli, Catharine I Paules

**Affiliations:** Penn State College of Medicine, Hershey, Pennsylvania; Penn State College of Medicine, Hershey, Pennsylvania; Penn State College of Medicine, Hershey, Pennsylvania; Penn State College of Medicine, Hershey, Pennsylvania; Penn State Health Milton S. Hershey Medical Center, Hershey, Pennsylvania; Penn State College of Medicine, Hershey, Pennsylvania; Penn State College of Medicine, Hershey, Pennsylvania; Penn State Health Milton S. Hershey Medical Center, Hershey, Pennsylvania

## Abstract

**Background:**

Emergency use of tixagevimab-cilgavimab was based on data collected prior to the emergence of SARS-CoV-2 omicron variant and subvariants. Dosing recommendations changed twice based on neutralization data alone, leading to patients receiving different doses at varying intervals. We provide real-world data on efficacy of tixagevimab-cilgavimab in immunocompromised patients throughout 2022, including the incidence and timing of infection based on dosing scheme.

**Methods:**

Charts of 471 patients who received and 126 patients who declined administration of tixagevimab-cilgavimab were analyzed through December 14^th^, 2022. We evaluated incidence of SARS-CoV2, severity, risk factors and vaccination status. For analysis, we grouped patients with similar dosing: Group A received one initial dose of 150mg/150mg of tixagevimab-cilgavimab, Group B received at least 300 mg/300 mg of tixagevimab-cilgavimab before July 1^st^, and Group C patients were up to date with the FDA dosing guidelines.

**Results:**

The tixagevimab-cilgavimab group had higher incidence of stem cell transplant or CAR T-cell therapy (115/471, 24% compared to 18/126, 14% p < 0.01) and had higher vaccination status (411/471, 89% compared to 37/126, 29%, p < 0.0001). Patients that received tixagevimab-cilgavimab had a trend towards higher incidence of SARS-CoV-2 infection (70/471, 15% compared to 11/126, 9%; p = 0.08). Incidence of SARS-CoV-2 was similar amongst all dosing groups (6/21, 29% in Group A; 37/251, 15% in Group B, and 27/199, 14% in Group C, p = 0.188). Group C experienced the most infections in November and early December 2022.

**Figure 1.**
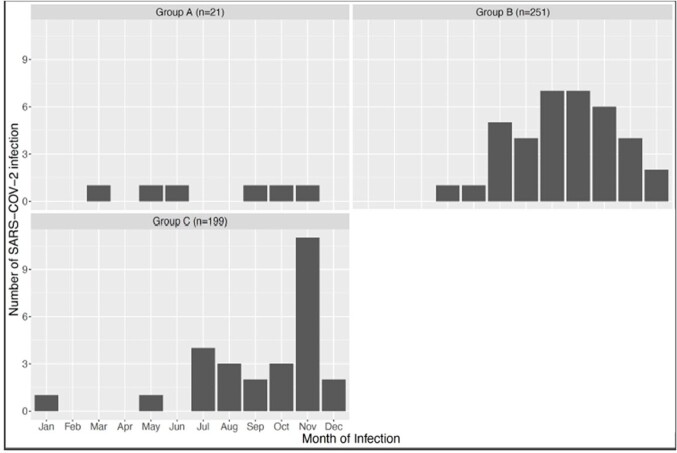
Number of SARS-CoV-2 infections per month based on dosing regimen

Group A patients received one initial dose of 150 mg/150mg of tixagevimab-cilgavimab. Group B patients received at least 300 mg/300 mg of tixagevimab-cilgavimab before July 1st, 2022 . Group C patients were up to date with the FDA dosing guidelines.

**Conclusion:**

The tixagevimab-cilgavimab group demonstrated increased incidence of SARS-CoV-2. This may reflect increased healthcare system utilization, as most infections were self-reported in this study. Those with the FDA recommended dose were most likely to develop SARS-CoV-2 in November and December 2022 when circulating variants were not neutralized by tixagevimab-cilgavimab in vitro. This suggests in vitro neutralization data correlates with clinical efficacy.

**Disclosures:**

**All Authors**: No reported disclosures

